# Comparison of Lymphocyte–CRP Ratio to Conventional Inflammatory Markers for Predicting Clinical Outcomes in COVID-19

**DOI:** 10.3390/jpm13060909

**Published:** 2023-05-29

**Authors:** Alexander Liu, Robert Hammond, Kenneth Chan, Chukwugozie Chukwuenweniwe, Rebecca Johnson, Duaa Khair, Eleanor Duck, Oluwaseun Olubodun, Kristian Barwick, Winston Banya, James Stirrup, Peter D. Donnelly, Juan Carlos Kaski, Anthony R. M. Coates

**Affiliations:** 1 School of Medicine, University of St Andrews, St Andrews KY16 9TF, UK; aql1@st-andrews.ac.uk (A.L.); rjhh@st-andrews.ac.uk (R.H.); pdd21@st-andrews.ac.uk (P.D.D.); 2 Royal Berkshire NHS Foundation Trust, Reading RG1 5AN, UK; kennethchan1@nhs.net (K.C.); dr.chuk@doctors.org.uk (C.C.); beckyljohnson@live.co.uk (R.J.); duaa_khair@hotmail.com (D.K.); eleanor.duck@gmail.com (E.D.); seun.r.olubodun@gmail.com (O.O.); kristianbarwick@outlook.com (K.B.); jimstirrup@hotmail.com (J.S.); 3 Royal Brompton Hospital, London SW3 6NP, UK; w.banya@rbht.nhs.uk; 4 Molecular and Clinical Sciences Research Institute, St George’s University of London, London SW17 0QT, UK; jkaski@sgul.ac.uk; 5 Institute of Infection and Immunity, St George’s University of London, London SW17 0QT, UK

**Keywords:** lymphocyte–CRP ratio, coronavirus disease 19, C-reactive protein, risk stratification, prognostic risk

## Abstract

**Background**: In COVID-19 patients, lymphocyte–CRP ratio (LCR) is a promising biomarker for predicting adverse clinical outcomes. How well LCR performs compared to conventional inflammatory markers for prognosticating COVID-19 patients remains unclear, which hinders the clinical translation of this novel biomarker. **Methods:** In a cohort of COVID-19 inpatients, we characterised the clinical applicability of LCR by comparing its prognostic value against conventional inflammatory markers for predicting inpatient mortality and a composite of mortality, invasive/non-invasive ventilation and intensive care unit admissions. **Results:** Of the 413 COVID-19 patients, 100 (24%) patients suffered inpatient mortality. On Receiver Operating Characteristics analysis, LCR performed similarly to CRP for predicting mortality (AUC 0.74 vs. 0.71, *p* = 0.049) and the composite endpoint (AUC 0.76 vs. 0.76, *p* = 0.812). LCR outperformed lymphocyte counts (AUC 0.74 vs. 0.66, *p* = 0.002), platelet counts (AUC 0.74 vs. 0.61, *p* = 0.003) and white cell counts (AUC 0.74 vs. 0.54, *p* < 0.001) for predicting mortality. On Kaplan–Meier analysis, patients with a low LCR (below a 58 cut-off) had worse inpatient survival than patients with other LCR values (*p* < 0.001). **Conclusion**: LCR appears comparable to CRP, but outperformed other inflammatory markers, for prognosticating COVID-19 patients. Further studies are required to improve the diagnostic value of LCR to facilitate clinical translation.

## 1. Introduction

In patients with acute coronavirus-19 (COVID-19), serum inflammatory markers play a major role in guiding clinical decision making [1,2]. C-reactive protein (CRP) is an established inflammatory marker and raised CRP levels are linked to increased disease severity and mortality risk in COVID-19 patients [1,2]. However, CRP reflects global innate immunity activation rather than informing specifically about the interactions between viral infections and adaptive immunity [1,2], and thus may not provide a comprehensive assessment of COVID-19 infections [1,2]. Other conventional inflammatory markers such as white cell counts (WCC), lymphocyte counts and platelet counts also have reported prognostic value in COVID-19 [3,4,5]. However, these markers are non-specific for viral infections, which limits their ability to provide a comprehensive assessment of the inflammatory response in COVID-19 [3,4,5]. 

Lymphocyte–CRP ratio (LCR) is a novel inflammatory index that has the potential to assess changes in both innate and adaptive immunity, and thus may provide a more comprehensive assessment of inflammation in viral infections [6,7]. LCR can be derived using routine blood results and has recently been shown to have prognostic value in COVID-19 [8,9,10,11,12,13]. Several reports have shown that a reduced LCR value is linked with an increased risk of invasive ventilation requirement, intensive care unit (ITU) admission and mortality [8,9,10,11,12,13]. Despite the recent evidence on LCR, it has not been compared to conventional inflammatory markers for predicting mortality and other serious adverse clinical outcomes in COVID-19 patients, which limits the potential introduction of LCR into clinical practice [8,9,10,11,12,13]. We set out to perform a head-to-head comparison of LCR against conventional inflammatory markers for the purpose of prognosticating COVID-19 patients. 

## 2. Materials and Methods

### 2.1. Study Subjects

**Figure 1:**
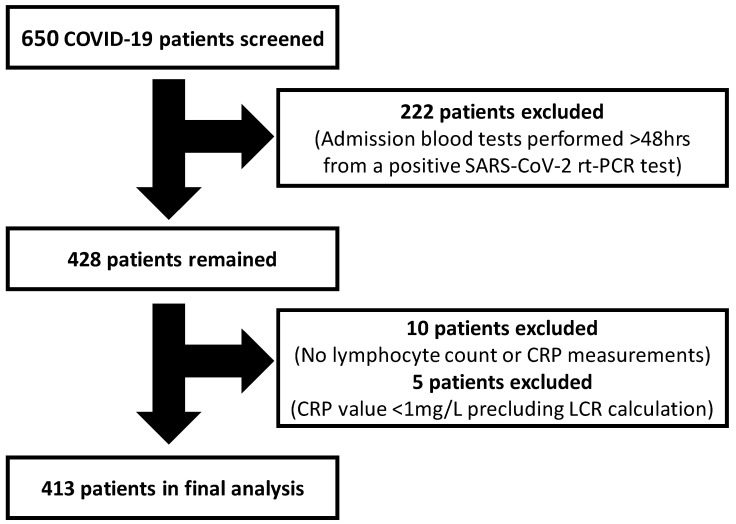
Flowchart illustrating the study patient screening and selection process. CRP: C-reactive protein; LCR: lymphocyte–CRP ratio; rt-PCR: real-time reverse transcriptase polymerase chain reaction.

This study included consecutive adult patients (18 years or older) with laboratory-confirmed COVID-19 admitted to the Royal Berkshire NHS Foundation Trust (UK) between 14 March 2020 and 9 May 2020. COVID-19 was diagnosed using real-time reverse transcriptase polymerase chain reaction (rt-PCR) testing of SARS-CoV-2 by nasopharyngeal swabs. Patients were excluded if they (i) had admission blood tests >48 h from their positive SARS-CoV-2 rt-PCR test (*n* = 222); (ii) did not undergo lymphocyte count or serum CRP measurement on admission (*n* = 10); or (iii) had documented unmeasurable CRP levels at <1 mg/L (*n* = 5) which precludes reliable LCR calculation. In total, 413 patients were included in the final analysis. The patient screening and selection process is illustrated in Figure 1. This study was granted COVID-19 Fast-Track Approval by the Health Research Authority (HRA) and Health and Care Research Wales (HCRW), UK.

### 2.2. Data Collection

Demographics data, clinical symptoms and laboratory test results were collected by a team of investigators (AL, KC, CC, RJ, DK, ED, OO and KB) according to a standardised data collection protocol and spreadsheet template. Each investigator was allocated a proportion of patients to collect data on. To ensure familiarity with data collection, the investigators were first asked to collect a sample dataset of ten cases. These were validated against the medical records by an independent observer. Upon satisfactory completion of the trial process, the investigators were asked to complete the data collection. To further ensure accuracy of the dataset, samples of the data were validated again by two observers (AL and KC) against the medical records, independent of the other data collectors. All observers were clinicians working in the COVID-19 frontline at the time the study was performed. 

### 2.3. Study Endpoints

The primary endpoint was inpatient mortality related to acute COVID-19. The secondary endpoints were defined as follows: (1) a composite of inpatient mortality, requirement for non-invasive ventilation (NIV), intubation/mechanical ventilation and intensive care unit (ITU) admission related to acute COVID-19; and (2) individual endpoints detailed in the composite endpoint. 

The composite endpoint was chosen to test the predictive values of LCR and conventional inflammatory markers for a range of clinically important adverse outcomes in COVID-19. It was important to include a composite endpoint to test the ability of LCR and CRP to rule in or rule out all possible major adverse outcomes associated with COVID-19 to aid clinical risk stratification and potential admission/discharge decisions. 

LCR was calculated using the following formula: lymphocyte count (number/μL) divided by CRP (mg/dL), as previously described [6]. 

### 2.4. Statistical Analysis 

Data were checked for normality using the Kolmogorov–Smirnov test. Parametric data were expressed as mean (standard deviation) and non-parametric data were expressed as median (inter-quartile range). Continuous data were compared using the Mann–Whitney test. Categorical data were compared using the Chi-squared test and where necessary in one case using the Fisher Exact test. Receiver Operator Characteristics (ROC) analysis was performed to assess the diagnostic performance of LCR and conventional inflammatory markers for predicting inpatient mortality and composite endpoints in COVID-19 patients. Where appropriate, area under the ROC curve (AUC) was presented with a 95% confidence interval. Kaplan–Meier curves were used to assess inpatient survival in COVID-19 patients and compared using the Logrank test. *p* < 0.05 denotes statistical significance. Statistical analysis was performed by AL (MedCalc; Version 12.7.8.0) and independently validated by WB, who is a medical statistician (Stata; Basic Edition version 17.0, Statacorp LLC, College Station, TX, USA). 

## 3. Results

### 3.1. Baseline Patient Characteristics

Of the 413 COVID-19 patients (median age 70 years (56–82); 58% males) in the study, there were 313 (76%) survivors and 100 (24%) non-survivors (Table 1). Non-survivors were older and presented with a lower prevalence of chest pain and fever compared to survivors (Table 1). Non-survivors had a lower proportion of asthmatic patients but a greater burden of atrial fibrillation, ischaemic heart disease, chronic kidney disease and chronic obstructive airways disease compared to survivors (Table 1). Other symptomology, co-morbidities and the medication history were similar between the two patient groups (Table 1).

### 3.2. Blood Results and Clinical Outcomes

**Figure 2:**
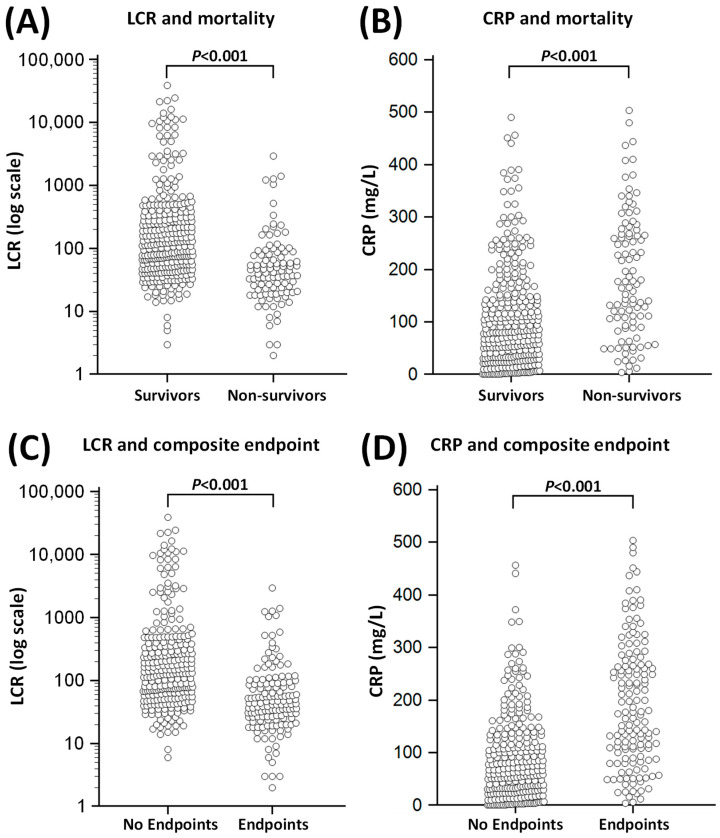
Relationship of C-reactive protein (CRP) and lymphocyte CRP ratio (LCR) with inpatient mortality (Panels (**A**) and (**B**)) and composite endpoint (Panels (**C**) and (**D**)) in acute COVID-19 patients. The composite endpoint included inpatient mortality, requirement for non-invasive ventilation, intubation/mechanical ventilation and/or intensive care unit (ITU) admissions. Each point represents data from a single COVID-19 patient.

Non-survivors had lower LCR (42 (21–84) vs. 119 (51–351), *p* < 0.001, Figure 2), lymphocyte counts (0.67 × 10^9^/L (0.45–1.00) vs. 0.94 × 10^9^/L (0.65–1.36), *p* < 0.001, Table 2) and platelet counts (188 × 10^9^/L (143–271) vs. 224 × 10^9^/L (178–289), *p* < 0.001, Table 2) compared to survivors. Conversely, non-survivors had higher CRP (169 mg/L (92–269) vs. 81 mg/L (33–152), *p* < 0.001, Figure 2) and serum creatinine (118 µmol/L (80–173) vs. 85 µmol/L (66–112), *p* < 0.001, Table 2) compared to survivors. 

NIV requirement was more common in non-survivors compared to survivors (27% vs. 11%, respectively, *p* < 0.001), whilst the prevalence of intubation and ITU admissions was similar between the two patient groups (Table 2).

### 3.3. Prognostic Data

**Figure 3:**
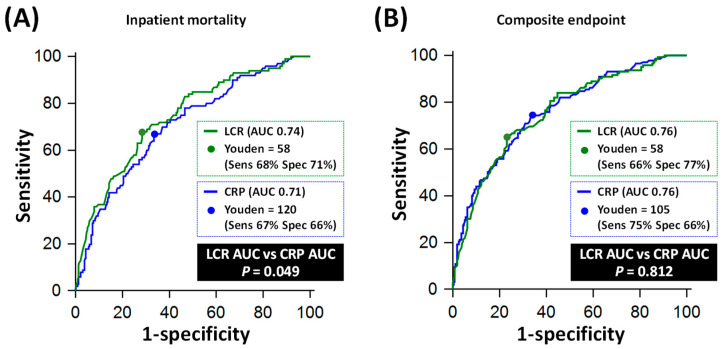
Comparative diagnostic performance of C-reactive protein (CRP) and lymphocyte–CRP ratio (LCR) for inpatient mortality and composite endpoint. Panel (**A**) shows the Receiver Operating Characteristics (ROC) curves of LCR and CRP for predicting inpatient mortality. Panel (**B**) shows the ROC curves of LCR and CRP for predicting a composite of inpatient mortality, requirement for non-invasive ventilation (NIV), intubation/mechanical ventilation and/or intensive care unit (ITU) admission. AUC: area under the ROC curve.

On ROC analysis, LCR (AUC 0.74, 95% CI: 0.70–0.78) performed similarly to CRP (AUC 0.71. 95% CI: 0.66–0.75) for predicting inpatient mortality, *p* = 0.049 (Figure 3A). An LCR cut-off of 58 yielded a sensitivity of 68% (95% CI: 58–77%) and a specificity of 71% (95% CI: 66–76%), whilst a CRP cut-off of 120 mg/L yielded a sensitivity of 67% (95% CI: 57–76%) and a specificity of 67% (95% CI: 61–72%) for predicting mortality (Table 3). 

For predicting a composite of mortality, requirement of NIV, intubation/mechanical ventilation and/or ITU admission, LCR (AUC 0.76, 95% CI: 0.71–0.80) also performed similarly to CRP (AUC 0.76, 95% CI: 0.71–0.80) on ROC analysis, *p* = 0.812 (Figure 3B). An LCR cut-off of 58 yielded a sensitivity of 66% (95% CI: 57–73%) and a specificity of 77% (95% CI: 71–81%), whilst a CRP cut-off of 105 mg/L yielded a sensitivity of 75% (95% CI: 67–81%) and a specificity of 66% (95% CI: 60–71%) for predicting the composite endpoint (Table 3). 

**Figure 4:**
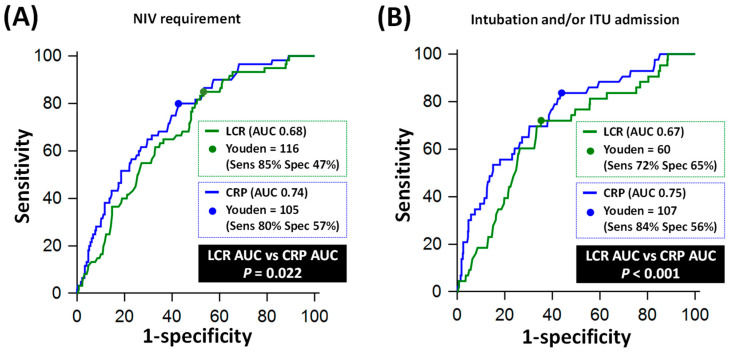
Comparative diagnostic performance of C-reactive protein (CRP) and lymphocyte CRP ratio (LCR) for individual non-mortality endpoints. Panel (**A**) shows the Receiver Operating Characteristics (ROC) curves of LCR and CRP for predicting requirement for non-invasive ventilation (NIV). Panel (**B**) shows the ROC curves of LCR and CRP for predicting requirement for intubation/mechanical ventilation and/or intensive care unit (ITU) admission. AUC: area under the ROC curve.

In terms of individual non-mortality endpoints, CRP outperformed LCR for predicting NIV requirement (AUC 0.74 vs. 0.68, *p* = 0.022) and for predicting intubation/ventilation and/or ITU admission (AUC 0.75 vs. 0.67, *p* < 0.001, Figure 4). 

**Figure 5:**
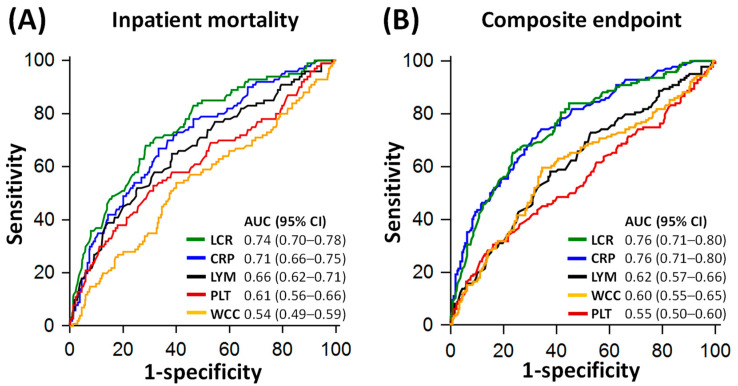
Comparative diagnostic performance of inflammatory markers for inpatient mortality (Panel (**A**)) and composite endpoint (Panel (**B**)). Composite endpoint included inpatient mortality, requirement for non-invasive ventilation (NIV), intubation/mechanical ventilation and/or intensive care unit (ITU) admission. AUC: area under the ROC curve; CI: confidence interval; CRP: C-reactive protein; LCR: lymphocyte CRP ratio; LYM: lymphocyte counts; PLT: platelet count; WCC: white cell count.

Compared to other inflammatory markers, LCR significantly outperformed lymphocyte counts (AUC 0.74 vs. 0.66, *p* = 0.002), platelet counts (AUC 0.74 vs. 0.61, *p* = 0.003) and WCC (AUC 0.74 vs. 0.54, *p* < 0.001) for predicting inpatient mortality (Figure 5). Whilst CRP was superior to platelet counts (*p* = 0.043) and WCC (*p* < 0.001) for the same purpose, CRP performed similarly to lymphocyte counts (AUC 0.71 vs. 0.66, *p* = 0.283) for predicting inpatient mortality (Figure 5). For predicting composite endpoints, both LCR and CRP significantly outperformed lymphocyte counts, platelet counts and WCC (all *p* < 0.001). 

### 3.4. Survival Analysis

**Figure 6:**
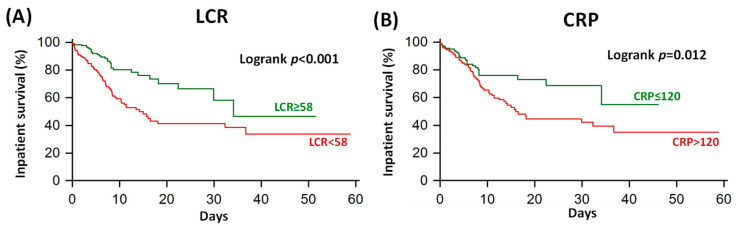
Kaplan–Meier inpatient 60-day survival analysis of lymphocyte–CRP ratio (LCR; Panel (**A**)) and CRP (Panel (**B**)). The Youden-point thresholds were derived from the Receiver Operating Characteristics curves displayed in Figure 3. CRP: C-reactive protein.

On Kaplan–Meier analysis, COVID-19 patients with LCR below 58 (optimal cut-off defined on ROC analysis) had impaired inpatient survival compared to patients with other LCR values, *p* < 0.001 (Figure 6A). Similarly, patients with CRP above 120 mg/L had impaired inpatient survival compared to patients with other CRP values, *p* = 0.012 (Figure 6B). 

## 4. Discussion

This study is the first to directly compare LCR against conventional inflammatory markers in a UK population of acute COVID-19 patients for predicting mortality and other severe adverse clinical outcomes. The main findings are that (i) LCR was comparable to CRP for predicting inpatient mortality and a composite of mortality, requirement for NIV, intubation/mechanical ventilation and ITU admission; (ii) CRP outperformed LCR for individual non-mortality endpoints; iii) LCR was superior to WCC, lymphocyte counts and platelet counts for predicting mortality and non-mortality endpoints; and (iv) patients with LCR <58 (cut-off derived from ROC) had worse inpatient survival compared to patients with other LCR values. LCR shows promise as a novel combination biomarker in acute COVID-19 and should be prospectively validated in a larger and multi-centre study. 

### 4.1. LCR: From Cancer to Coronavirus

The rationale behind the development of LCR as a combination biomarker has two aspects. Firstly, although CRP is an established marker in the management of infections [14], it is non-specific for viral infections [14], and the notion of a new and potentially more comprehensive biomarker, such as LCR, for risk stratifying COVID-19 patients is highly desirable. Secondly, LCR has already shown promise for prognosticating patients with gastrointestinal malignancies, where it is believed to act as a surrogate marker for host–tumour immune interactions [6,7]. Since lymphocytes play an important role in combating both cancer and viral infections [15], LCR, already useful in cancer patients [6], may translate itself into a potential COVID-19 prognosticator.

As an inflammatory index, LCR exists mathematically as a function of both CRP levels and lymphocyte counts [9]. Since both elevated CRP levels [1,2] and reduced lymphocyte counts [4] are reportedly linked to an adverse prognosis in COVID-19 patients [1,2,4], LCR could potentially exploit the prognostic value of both markers in either a synergistic or additive fashion [9,16]. From a physiological viewpoint, lymphocytes are key mediators of adaptive immunity [17] whilst CRP influences innate immunity and partly adaptive immunity [18]. The combination of lymphocyte count and CRP as a single biomarker may therefore provide a more comprehensive assessment of the overall inflammatory response in acute COVID-19. 

The ability to predict inpatient prognosis using biomarkers is important for guiding clinical management in COVID-19 patients [19]. Although several observational studies have suggested that LCR can predict inpatient mortality and COVID-19 disease severity [8,9,10,11,12,13], most of these studies have been based on relatively small sample sizes [9,10,11,12,13]. Tonduangu and colleagues [8] demonstrated in a multi-centre study of 1035 patients that the ratio between lymphocyte and CRP achieved reasonable diagnostic performance for predicting severe COVID-19 (AUC 0.679; cut-off 78.3; sensitivity 79%, specificity 47%) and mortality (AUC 0.607; cut-off 159.4; sensitivity 48%, specificity 70%) [8]. The diagnostic performance of LCR in our study appears slightly higher, which may be related to differences in characteristics between the study populations [8]. A prospective validation of LCR in a larger all-comers COVID-19 population would address these inter-study differences. 

### 4.2. LCR: A Marker of Potential Incremental Value

A recent study showed that CRP and novel markers such as CRP-to-lymphocyte ratio (CLR), neutrophil-to-lymphocyte ratio (NLR) and platelet-to-lymphocyte ratio (PLR) can all predict oxygen requirement in COVID-19 patients [20]. However, missing from the literature until now has been an adjudication of the relative performance of LCR as compared to CRP for predicting mortality and other serious adverse outcomes (such as intubation and ICU requirement) in acute COVID-19; this study provides such head-to-head comparison. Since CRP is the established and widely available inflammatory biomarker in clinical practice [1,2,12], any new inflammatory marker should be compared against CRP as a benchmark before being considered for clinical translation. 

By achieving respectable diagnostic performance for predicting a composite of mortality, NIV, intubation and ITU admission in COVID-19 patients, LCR appears to be useful in assessing the overall risk of any one or more of the adverse outcomes occurring. If prospectively validated, LCR could potentially classify patients into a low-risk group (no adverse outcomes) vs. a high-risk group (one or more negative outcomes). This could be useful throughout the healthcare system in a number of scenarios: (1) to assist medical staff in the emergency department or acute medical units in deciding whether to admit or discharge newly diagnosed COVID-19 patients; (2) to assist inpatient medical staff to decide on therapeutic allocation for admitted COVID-19 patients; and (3) to potentially assist primary care practitioners to decide on whether a patient requires hospital admission, which should also be validated.

The respectable prognostic value of LCR in the study exemplified the potential of the “combination biomarker” concept for prognostication [16]. Whilst CRP and lymphocyte counts performed similarly in predicting mortality in the study population, by combining the two biomarkers into LCR, the resultant prognostic value for mortality appeared to have increased to a level slightly higher than each of its constituents. It remains unclear whether this is a synergistic or additive effect, since the effect size of the difference in AUC between CRP to LCR was small. Further work is needed to elucidate the mechanism that drove this effect. 

### 4.3. Limitations and Future Directions

The strength of this study lies in its relatively larger sample size compared to most previous studies on LCR, the completeness of the LCR and CRP data collection and the robust interrogation of their relative diagnostic performance. However, certain study limitations exist which give rise to potential future investigations to enrich our understanding of inflammatory processes in COVID-19. As a retrospective observational study, LCR would not have been used to influence treatment decisions, whilst CRP would have guided management. A prospective study comparing LCR against CRP would add further value in estimating their relative prognostic values in real-world practice. It was also not possible to segregate lymphocytes into subtypes such as T-cells, B-cells and natural killer cells [21], which might offer further insights into the effect of lymphopenia on prognosis [21]. Further, this study did not have access to data on non-routine inflammatory markers, such as interleukins [22], which might inform about host–viral interactions beyond routine blood tests. 

## 5. Conclusions

The results in this study suggest that LCR is comparable to CRP, but outperformed other inflammatory markers, for prognosticating COVID-19 patients. Further studies are required to improve the diagnostic value of LCR to facilitate its clinical translation.

## Figures and Tables

**Table 1:** Baseline patient characteristics.

	All Patients (*n* = 413)	Survivors (*n* = 313)	Non-Survivors (*n* = 100)	*p* Value
Age	70 (56–82)	66 (52–81)	79 (71–86)	<0.0001
Male	240 (58)	180 (58)	60 (60)	0.66
BMI	26 (22–30)	27 (22–30)	25 (21–30)	0.164
Symptoms				
Chest pain	45 (11)	40 (13)	5 (5)	0.030
Cough	257 (62)	199 (64)	58 (58)	0.317
Dyspnoea	250 (61)	184 (59)	66 (66)	0.199
Fatigue	106 (26)	78 (25)	28 (28)	0.539
Fever	219 (53)	177 (57)	42 (42)	0.011
Comorbidities				
Atrial fibrillation	61 (15)	37 (12)	24 (24)	0.003
Ischaemic heart disease	60 (15)	38 (12)	22 (22)	0.015
Heart failure	44 (11)	30 (10)	14 (14)	0.213
Hypertension	188 (46)	135 (43)	53 (53)	0.084
Diabetes	111 (27)	80 (26)	31 (31)	0.301
Dyslipidaemia	50 (12)	33 (11)	17 (17)	0.087
Smoker (current and ex)	119 (31)	81 (28)	38 (38)	0.077
CKD	99 (24)	66 (21)	33 (33)	0.016
COPD	47 (12)	26 (9)	21 (21)	0.001
Asthma	58 (14)	51 (16)	7 (7)	0.020
CVA/TIA	38 (9)	27 (9)	11 (11)	0.475
Dementia	56 (14)	39 (13)	17 (17)	0.248
Medications				
ACEi/ARB	105 (25)	76 (24)	29 (29)	0.345
Warfarin	19 (5)	11 (4)	8 (8)	0.095
DOAC	47 (11)	31 (10)	16 (16)	0.095
Aspirin	57 (14)	42 (14)	15 (15)	0.690
Statins	145 (35)	107 (34)	38 (38)	0.487

BMI: body mass index; CKD: chronic kidney disease; COPD: chronic obstructive pulmonary disease; CVA: cerebrovascular accident; ACEi: angiotensin converting enzyme inhibitor; ARB: angiotensin receptor blocker; DOAC: direct oral anticoagulant.

**Table 2:** Patient observations, laboratory results and complications.

	All Patients (*n* = 413)	Survivors (*n* = 313)	Non-Survivors (*n* = 100)	*p* Value
Observations on admission				
Temperature	37.1 (36.6–37.9)	37.1 (36.7–37.9)	37.1 (36.5–37.9)	0.389
SBP	129 ± 24	130 ± 24	124 ± 24	0.0305
DBP	74 ± 14	75 ± 14	70 ± 15	0.0007
Respiratory Rate	22 (18–26)	20 (18–24)	24 (20–28)	<0.001
Laboratory Results				
LCR	82 (41–264)	119 (51–351)	42 (21–83)	<0.001
Lymphocyte Count	0.90 (0.60–1.31)	0.94 (0.65–1.36)	0.67 (0.45–1.00)	<0.001
CRP	102 (41–187)	81 (33–152)	169 (92–269)	<0.001
Haemoglobin	127 (111–143)	129 (114–145)	121 (108–134)	<0.001
WCC	7.2 (5.3–10.1)	7.0 (5.3–10.0)	8.0 (5.2–11.5)	0.240
Platelet Count	216 (171–286)	224 (178–289)	188 (143–271)	<0.001
Sodium	138 (134–140)	138 (134–140)	138 (135–140)	0.733
Potassium	4.2 (3.9–4.5)	4.2 (3.9–4.5)	4.2 (3.8–4.7)	0.094
Creatinine	89 (67–128)	85 (66–112)	118 (80–173)	<0.001
Complications				
NIV requirement	60 (15)	33 (11)	27 (27)	<0.001
ITU admission	42 (10)	29 (9)	13 (13)	0.282
Intubation	24 (6)	16 (5)	8 (8)	0.283

SBP: systolic blood pressure; DBP: diastolic blood pressure; WCC: white cell count; CRP: C-reactive protein; LCR: lymphocyte–CRP ratio; NIV: non-invasive ventilation; ITU: intensive care unit.

**Table 3:** Diagnostic performance of LCR and CRP for predicting mortality and composite endpoints in acute COVID-19 patients.

	For Predicting Mortality		For Predicting Composite Endpoint	
	LCR	CRP (mg/L)	LCR	CRP (mg/L)
Optimal cut-off (Youden)	58	120	58	105
Sensitivity (95% CI)	68% (58–77)	67% (57–76)	66% (57–73)	75% (67–81)
Specificity (95% CI)	71% (66–76)	67% (61–72)	77% (71–81)	66% (60–71)
Positive LR (95% CI)	2.4 (1.9–3.0)	2.0 (1.6–2.5)	2.8 (2.2–3.6)	2.2 (1.8–2.6)
Negative LR (95% CI)	0.5 (0.3–0.6)	0.5 (0.4–0.7)	0.5 (0.4–0.6)	0.4 (0.3–0.5)
PPV (95% CI)	43% (35–51)	39% (32–47)	60% (52–68)	54% (47–61)
NPV (95% CI)	88% (83–91)	86% (81–90)	80% (75–85)	83% (77–88)

CI: confidence interval; CRP: C-reactive protein; LCR: lymphocyte–CRP ratio; NPV: negative predictive value; PPV: positive predictive values. Composite endpoints included inpatient mortality, requirement for non-invasive ventilation (NIV), intubation/mechanical ventilation and/or intensive care unit (ITU) admission.

## Data Availability

The study data are available upon reasonable request from the corresponding author.
